# Seaweed Value Chain Stakeholder Perspectives for Food and Environmental Safety Hazards

**DOI:** 10.3390/foods11101514

**Published:** 2022-05-23

**Authors:** Jennifer L. Banach, Sophie J. I. Koch, Yvette Hoffmans, Sander W. K. van den Burg

**Affiliations:** 1 Wageningen Food Safety Research (WFSR), Wageningen University & Research, 6700 AE Wageningen, The Netherlands; yvette.hoffmans@wur.nl; 2 Wageningen Economic Research, Wageningen University & Research, Droevendaalsesteeg 4, 6708 PB Wageningen, The Netherlands; sophie.koch@wur.nl (S.J.I.K.); sander.vandenburg@wur.nl (S.W.K.v.d.B.)

**Keywords:** monitor, mitigate, protocol, arsenic, iodine, pathogen, ecological impact, genetically modified species, non-indigenous species, sustainable harvesting

## Abstract

With a world population estimated at 10 billion people by 2050, the challenge to secure healthy and safe food is evident. Seaweed is a potential answer to this challenge. Expanding the use of seaweed in food systems requires an emphasis on safe practices to avoid adverse human health effects after consumption and irreversible damage to marine ecosystems. This study aims to evaluate relevant food safety and environmental safety hazards, monitoring measures, and mitigation strategies in the seaweed sector. For this study, a literature review, survey (*n* = 36), and interviews (*n* = 12) were conducted to identify hazards. The review and interviews aimed at pinpointing monitoring measures and mitigation strategies applied, while the survey revealed data gaps and further actions needed for the sector. Relevant food safety hazards include (inorganic) arsenic, iodine, and heavy metals, among others, such as pathogenic bacteria, while environmental hazards include environmental pathogens and parasites introduced into the ecosystem by domesticated seaweed, among others. Measures applied aim at preventing or mitigating hazards through good hygienic or manufacturing practices, food safety procedures or protocols, or pre-site farm selection. Although the future needs of the seaweed sector vary, for some, harmonized advice and protocols that align with a changing food system and hazard knowledge development as well as information on the benefits of seaweed and regulating climate and water quality may help.

## 1. Introduction

In order to meet the needs of a growing world population coming close to 10 billion people by 2050, food production should increase by an estimated 50% compared to current levels [1]. Although today’s world food production is enough to feed everyone, more than 800 million people face chronic hunger, whereas half of the present world’s population is either malnourished or obese. The production of increasingly nutritious food requires significantly more land and other inputs such as minerals, water, and energy. Yet, the natural resource base necessary to contribute to the global food needs is deteriorating, ecosystems are under stress, and biological diversity is declining around the globe [2]. For these reasons, there is an increasing interest in the potential of the seas to provide food for direct human consumption or feeding animals [3,4].

Due to a growing interest in seaweed, there have been developments in the market for seaweed production, especially considering its use for food and animal feed [5,6]. For instance, in the European algae sector, there are a reported 225 companies producing macroalgae, with France, Ireland, and Spain having the most macroalgae companies. European aquaculture production of *Saccharina latissima*, a seaweed with rich nutritional content for food and feed purposes, has the highest production volume (376 tonnes fresh weight (fw)) and the number of companies (26), followed by *Alaria esculenta* (107 tonnes fw and 16 companies), and *Ulva* sp. (50 tonnes fw and 10 companies) [5]. In Eastern Asia, there is a demand for seaweed as there it is frequently consumed as food [7,8,9]. In 2019, total seaweed production (both farmed and wild) in Asia contributed to 97.4% of the world’s production, with China ranked highest, making up 56.8% of the world’s production. Reported brown seaweed production was 16.4 million tonnes (in 2019), making up 47.3% of the world’s cultivation, with *Laminaria*/*Saccharina* cultivation totaling 12.2 million tonnes wet weight (ww), the majority of which was produced in China (11.0 million tonnes ww).

With the growth of the seaweed sector for food purposes, there is more attention towards safe practices, including food safety [10,11] and environmental safety [12,13,14] in the seaweed value chain. Although some hazards have been identified for food safety [10], currently, there is no overview of the industry relevance of these two safety domains. Reported data on current seaweed monitoring measures and/or mitigation strategies is limited. Monitoring could provide valuable information on the value chain and may support future seaweed growth in a safe way. An analysis of the relevant food and environmental concerns alongside monitoring and/or mitigating practices provides fundamental support for the next steps for sector growth, such as gaining more consumer trust and setting clear regulations to anticipate and mitigate hazards.

This study aims to evaluate relevant food safety and environmental safety hazards in the seaweed sector from various perspectives, including that from the industry. The main research questions addressed are:

1. What are the relevant food safety and environmental safety hazards for the seaweed sector?
2. Which monitoring measures and/or mitigation strategies are currently implemented for the relevant hazards?
3. Where are the gaps, and what further actions are needed?

The focus of this research is on edible seaweeds. Results were based on a literature review, an online survey, and stakeholder interviews. Given the reportedly large seaweed production figures in Eastern countries, such as China, this study also considered information in Standard Chinese.

Our data collection through the literature review, the survey, and the interviews aimed at answering all three questions. However, not all three data sources have the same focus on each question. In the discussion section, we discuss all collected data in detail, contributing to answers regarding all three questions with our own reflections.

## 2. Materials and Methods

### 2.1. Literature Review

#### 2.1.1. Food Safety

The literature review part of this study aimed to identify hazards in edible types of seaweed when consumed as food. Literature was searched during March 2021 in the bibliographic databases Scopus (https://www.scopus.com/home.uri, accessed on 24 March 2022) and Web of Science (https://clarivate.com/webofsciencegroup/solutions/web-of-science/, accessed on 24 March 2022) for the period of 2015–2021. The search terms were built up in three search strings:

1. “Seaweed”
2. “Food” OR “human consumption”
3. “Food safety” OR “hazard” OR “adverse effect” OR “risk”

These terms were searched in the abstract, title, and keywords of scientific articles. A three-tiered approach was used to select articles for full-text reading. First, the articles were downloaded into a reference management software package (EndNote X9, Clarivate^TM^, Philadelphia, PA, USA) and sorted into the categories ‘relevant,’ ‘not relevant,’ and ‘maybe relevant’ based on the inclusion of information on food safety hazards or substances that may exert an adverse effect on humans upon the consumption of seaweed. Second, the ‘relevant’ articles were divided into groups: metals, iodine, and other food safety hazards. Finally, the articles were retained after the first two steps and read in full. In addition, since the Chinese seaweed sector is more developed compared to that of Western/English-speaking countries, literature published in Standard Chinese or by Chinese researchers from 2015–2021 on food safety aspects of seaweed was reviewed. Potential hazards and major seaweed products in China were used as keywords to search the Chinese literature from April-May 2021, using the database China National Knowledge Infrastructure (CNKI) (https://www.cnki.net/, accessed on 24 March 2022). In total, 47 articles were reviewed.

#### 2.1.2. Environmental Safety

The literature review part of this study aimed to gather further insight on the environmental safety of seaweeds and potential hazards. References used in previous scientific reports that focused on seaweed farming were reviewed. From there, additional literature from 2015–2021 was selected. Search terms related to seaweed farming or production and seaweeds (*laminaria*, *S. latissima*) were used. When needed, literature was snowballed. Similarly, literature published in Standard Chinese or by Chinese researchers from 2015–2021 on environmental aspects of seaweed was reviewed with potential hazards, and major seaweed products in China were used as keywords to search the Chinese literature from April-May 2021, using the database China National Knowledge Infrastructure (CNKI) (https://www.cnki.net/, accessed on 24 March 2022). Finally, when required, personal experiences working directly in the seaweed sector and expertise on this topic were consulted to determine the relevancy of the papers retrieved.

#### 2.1.3. Data Collection

From the reviewed articles, hazards were grouped in Excel by type, i.e., chemical, physical, microbiological, or unknown. When available, the seaweed type along with the genus and species were included. When literature included the part of the chain where samples had been taken, this information was noted. In addition, other background information on the product (sample), country of origin, type of cultivation, the environment it was grown, and harvest period—including month and year collected—were recorded when reported in the literature. Finally, recommendations from the study, including practical recommendations, along with data gaps and the ranking (based on the source), were noted.

### 2.2. Survey

Based on the results of the literature review part of the study and expert input from the study team, a long list of 14 food safety and 22 environmental safety hazards was formulated. An online survey using Microsoft Forms was made to gain insight into the risk perception of all types of stakeholders involved in the seaweed sector. Questions were mainly close-ended and consisted of statements set on a five-point scale, where 1 represented a very small risk and 5 a very large risk. The potential impact of hazards was formulated as an open question to keep the survey at a feasible length. Questions to pinpoint current monitoring and/or mitigation performed and future sector needs were included, such as the use of protocols, standards, or certification schemes. The survey was available online from the end of June 2021 until mid-August 2021.

Some questions were targeted toward the type of respondent. The distribution of respondents was checked regularly during the survey collection, and additional measures to reach underrepresented groups were carried out. The Safe Seaweed Coalition network [15] was used to spread the survey, as well as professional social media accounts from project team members (e.g., LinkedIn), personal invitations by e-mail, and an online article via the Wageningen University and Research website [16]. To avoid bias via the personal e-mails, they were not only sent to selected researchers but to all colleagues working in the field.

### 2.3. Interviews

Stakeholder interviews were conducted to provide additional insight on hazards in seaweed as well as monitoring and/or mitigation performed and future perspectives for the seaweed industry. A list of potential interviewees, based on project members’ professional networks, was established. Networking tools such as LinkedIn were also consulted to compile the list. From this list, 21 companies, mainly located in Europe and Asia, were contacted for an interview. Companies selected were those that appeared to be or were closely related to seaweed producers and farmers in the seaweed value chain. An open interview guide was used to obtain background information on the interviewees (see Supplementary Materials). The interviews were scheduled for about 1 h. Following the interviews, the notes were summarized and shared with the interviewee for approval.

## 3. Results

### 3.1. Literature Review

From the evaluated articles on food safety and environmental safety, we identified 130 hazards, with roughly 73% regarded as food safety and 27% as environmental safety.

#### 3.1.1. Food Safety

Literature on seaweed identified several agents that may be seen as food safety hazards, including bacteria, viruses, pharmaceutically active substances, chemical elements, and persistent organic pollutants, along with hazards such as allergens, endocrine-disrupting compounds, pesticide residues, and marine toxins. According to the literature review, food safety hazards of concern for the seaweed sector include arsenic, iodine, and heavy metals such as cadmium, lead, and mercury. Allergens may be of further interest to monitor given limited data combined with the potential health risk to certain consumers. Information on mitigation strategies is not described abundantly in literature for several food safety hazards in seaweed. The food safety hazards arsenic and iodine have been subject to research in relation to the minimalization of their presence in seaweed. A summary of the possible mitigation of these two food safety hazards is below.

**Arsenic**

Especially for inorganic arsenic, mitigation strategies are needed to minimize the risk thereof in seaweed due to the potential for high concentrations of inorganic arsenic in seaweed and the toxicity thereof. A strategy mentioned in the literature that is seemingly effective in lowering arsenic concentrations is boiling seaweed. The practice of boiling (T = 90 °C, t = 5 min) and soaking in a solution with 2% NaCl previously showed a reduction in the concentration of inorganic arsenic in the seaweed *Sargassum fusiforme* [17]. Although not all studies showed a reduction of arsenic in seaweed (*Porphyra* in this example) [18], boiling may still be a valuable mitigation strategy. The boiling of *Porphyra* did not result in any significant change in concentrations of total arsenic but had a mitigation effect on other metals, such as copper, iron, and selenium [18].

**Iodine**

Iodine could be a risk to humans after the consumption of seaweed due to potentially high levels thereof in seaweed. The establishment of legal limits for iodine in seaweed is urged in order to lower the dietary intake of iodine from seaweed [19]. Communicating the levels of iodine in seaweed may help create awareness about the dietary exposure to iodine from seaweed [19]. Similar to arsenic, for iodine, the mitigation strategy of boiling can lower the iodine concentrations in seaweed. A reduction of approximately 90% in iodine content was achieved by boiling brown seaweed (kelp) for 20 min [18]. Furthermore, practices such as drying and frying were also demonstrated to be effective in the reduction of iodine in *S. latissima* (processing parameters were unspecified). Due to the water solubility of iodine, part of the iodine content (~25%) in the seaweed is evaporated during drying. Frying results in reductions varying from 25% to 80% in iodine content in *S. latissima* [20]. Another high reduction of iodine (by 68%) was seen in the seaweed *A. esculenta* when it was first dehydrated for 72 h, then rehydrated again for 24 h, and finally boiled for 20 min. The application of the same treatment to the seaweed *P.* *esculenta* resulted in a lower (by approximately 25%) reduction of iodine [21].

#### 3.1.2. Environmental Safety

The structure of this section differs from the one on food safety hazards because the literature did not suggest a certain number of hazards clearly posing a higher risk than others. This section refers to the hazards most referred to in more detail and some lesser-known hazards in less detail.

Seaweed is commonly cultivated on lines, mostly comprised of a mixture of synthetic polymer rope (e.g., polypropylene) [22], as there are not yet biodegradable solutions. When seaweed is grown, the ropes are pulled out of the water, and the seaweed is cut off. Depending on how close it is to the rope, more or fewer fibers are being shredded off and enter the harvested seaweed batch or the surrounding water body). However, besides those lines, there can also be moorings and floats, rendering the whole system more complex and offering possibilities for litter if there is no proper site husbandry and management [22]. All systems comprise a mixture of moorings, lines, and floats with varying degrees of complexity. Not only plastic from the ropes but also plastic and litter already present in the water can increase levels of plastic in marine food webs [23]. The amount of litter and synthetic compounds found in the water are an influencing factor; of course, sporadic litter or synthetic pollution in a water body of good quality is less of a threat than if they have become chronic and persistent, which in itself may be regulated by the authorities. The nature and shape of the litter (e.g., microplastics are more difficult to detect and remove) can also be relevant when determining the level of threat it poses [24]. Furthermore, microcracking through weathering and degradation of plastic increases the number of microparticles in the water [23] (either from the beach into the sea or breaking apart in the water). In order to avoid litter from the farm entering the ecosystem, preventative measures are again recommended: using biodegradable material and good site husbandry, ensuring cultivation systems are fit for purpose [22], as well as regular maintenance and replacement of loose or damaged material [14].

A seaweed farm can take up too many nutrients and risk local depletion and/or create too much shadow, both limiting the growth of, for example, primary production (phytoplankton) [25]. To minimize the negative impact through nutrient uptake and shading from the farm onto the environment, the choice of the farm can play a big role in the prevention of certain hazards [22,26,27]. Negative effects of shading could be avoided if it was chosen not to farm in well-vegetated areas or areas frequented by protected and vulnerable communities [22] or if the chosen site for a farm is rather in eutrophic conditions than in water bodies with limited nutrients [27].

Another relevant hazard to the environment is the disturbance of the endemic marine species. Cultivation sites will have localized increased noise disturbance through boat and vessel engines or even the cultivation system equipment such as machinery for installation, maintenance, seeding, and harvesting [22]. Noise disturbance can depend on the size of the farm and how far away marine fauna is. It is considered that the noise disturbance is proportionate to the scale of operation [22]. The negative effect of this hazard can be limited by selecting a site without vulnerable species [22].

Even if the exact consequences are unknown of introducing genetically and phenotypically different seaweed cultivars to the naturally occurring populations, there are potentially significant consequences for the environment assumed. This could be through hybridization or direct competition with wild populations [28,29,30]. Cultivated seaweed will have gone through a human-imposed shift in its reproductive strategy, for example, to favor self-fertilization or vegetative reproduction. This may narrow their genetic diversity, with the results potentially making them more susceptible to environmental changes and disease [30,31]. With large-scale cultivations, it is unavoidable that this reproductive material of the domesticated stands will be released into the surrounding ecosystem and come into contact with locally seaweed stands [30]. Releasing reproductive material was assessed to have an instantaneous low overall impact risk but is relatively important to a particular ecosystem component; introducing genetically modified species was assessed to have the same impact but in addition also in the long run. The input of non-indigenous species was assessed to have a high overall impact risk, instantaneous as well as for the long term, and furthermore, a long-term low-impact risk that is relatively important to a particular ecosystem component [14].

In order to prevent the introduction of unwanted genetically modified species or non-indigenous species, preventive measures are a recommended mitigation strategy: using locally sourced reproductive material will maintain the genetic integrity of local communities and can be a very efficient mitigation strategy to avoid the introduction and spread of unwanted domesticated seaweed strands [22,30].

The farm structure itself can provide opportunities for invasive species to settle, with potential negative consequences such as the loss of biodiversity (possibly even including the disappearance of indigenous species), the introduction of new diseases and economic damage, and, in certain or in a location where seagrass and kelp forests are at the base of the ecosystem, there could even be competition for nutrients and space [14]. The system structure itself can also be a barrier to species movement or damage the seabed [14,22]. The disturbance of the seabed depends on how many anchors and chains are put in and for how long. Some farms, for example, do not put anything permanent in. Examples of hazards that have a high impact risk due to their persistent pressure even after decommissioning are the introduction of non-synthetic substances and compounds, such as heavy metals and hydrocarbons that cause pollution [14].

Further hazards are the potential reduction in wave energy or water flow changes through the seaweed farm, entanglement of fauna in the farm structure, collision of fauna with the boats or farm structure, change in siltation, the input of microbial pathogens, and input of organic matter [14,22].

### 3.2. Survey

In total, 35 respondents completed one or more parts of the survey. Of the 35 respondents, 18 worked directly in the seaweed industry, as a cultivator, manager, etc., while 17 did not work directly in the industry yet rather, in academia, for governmental or non-governmental organizations, had a general interest in seaweed, or processed seaweed into other products (*n* = 11). Respondents had the option to omit the food safety or environmental safety sections due to, for instance, lack of knowledge or interest. Almost half of the respondents (48%) operate from either the UK (32%) or other countries in the EU (16%), while the rest (52%) come from North America (19%), Asia (10%), or worked on 2 or more continents (23%). Furthermore, over a third of companies (38%) have a local (10%) or national (28%) scope, while 44% reach a market outside of their national borders. Of that 44%, 13% have an international/regional scope, and 31% have a global scope. The results from the remaining 17% could not be used. Since there was no means to properly translate and distribute the survey to non-English-speaking persons, the study had mainly reached European (*n* = 15) and North American (*n* = 6) seaweed, plus seven respondents that worked on at least one of those continents. The following sections describe the survey results on the relevant hazards for the seaweed sector and data gaps/further actions needed for, respectively, food safety and environmental safety.

#### 3.2.1. Food Safety

Twenty-seven respondents completed the food safety section of the survey and scored the fourteen hazards. Respondents were again categorized into two groups, those working directly in the seaweed industry (*n* = 16) and those not working directly in the industry (*n* = 11). After providing scores for the 14 listed hazards (Table 1), respondents were asked if they considered other hazards as a risk to seaweed as food, of which 30% responded “yes”. Also, separate open questions were posed for biological, chemical, and physical hazards, the results of which are shown below. Respondents could provide multiple hazards in their open response; hence, the number of hazards exceeds the number of respondents.

Results showed that the industry and non-industry stakeholder groups both ranked arsenic highest, 3.4 and 4.0, respectively, while the non-industry stakeholder group scored iodine higher (3.4) than the industry stakeholder group (2.6). When considering all respondents, arsenic and iodine were the highest, while PAHs and radionuclides were the lowest (Table 1). The presence of persistent organic pollutants (POPs) such as dioxins and polychlorinated biphenyls (PCBs) was generally higher for the industry stakeholder group (both 2.7) than the non-industry stakeholder group (2.4 and 2.3, respectively). Seaweed can absorb other toxic substances such as heavy metals (cadmium, mercury, lead), PAHs, and organochlorine pesticides and may pose a risk to consumers if present in sufficient amounts. This can be inferred from the rather close scores (about 2–3) for these types of hazards. The results from the open questions showed that 14 of the 27 respondents (52%) provided additional chemical hazards that may pose a risk, thus supporting this thought.

From the open questions, heavy metals, such as cadmium, lead, or mercury, were mentioned by 9 of the 27 respondents (33%), of which four respondents scored this group a 5. One respondent elaborated that cadmium is of far less concern than inorganic arsenic. Furthermore, allergens that can be associated with seaweed (such as shellfish and crustaceans) were mentioned by 5 of the 27 respondents (19%). Two respondents, both from non-industry, answered halogenated compounds (bromoform, etc.), of which one also mentioned secondary metabolites thereof specifically. Other chemical hazards mentioned were kainic acid, bromoform compounds, acidification of the ocean (to prevent algal growth), and contaminants in the water due to human populations nearby. Given the additional responses from the open question, heavy metals (cadmium, lead, and mercury) remain an important group to monitor and mitigate, along with arsenic and iodine, given their potential risks. Allergens may deserve more attention as they can pose a health risk to certain consumers.

Similarly, concerns with biological hazards such as pathogenic bacteria and viruses were scored higher by industry than non-industry (Table 1). An explanation for this difference may be due to stricter microbiological requirements or standards for the industry to be able to sell further in the value chain (e.g., from business-to-business or business-to-consumer). From the open questions, 4 of the 27 respondents (15%) provided additional biological hazards that may pose a risk. Responses included *E. coli* (*n* = 2), as well as yeast, fungi, invasive species, and absorption of other toxic residues (each *n* = 1). The *E. coli* serotype was unspecified. Likewise, the pathogenic nature of the *E. coli* was not indicated, which is important to know as there are nonpathogenic *E. coli* that can be used as indicator microorganisms. Most of these examples are presumably related to the microbiology quality of the seaweed and can be associated with the shelf life of a product, the latter of which is out of the scope of this study.

In the list of 14 hazards, one physical hazard—plastic—was mentioned and scored similarly by industry stakeholders (2.6) and non-industry stakeholders (2.5) (Table 1). In the open questions, 5 of the 27 respondents (19%) provided additional physical hazards that may pose a risk. Responses included the presence of stones and shells; storm surges; pieces of fish and shellfish (which could be related to allergenic issues); torn plastic or rope that contaminate the seaweed; and biofouling. The latter one was perceived as high risk (i.e., was scored a 5).

To the question if the seaweed industry was to further develop in the future and if the hazards would then become a bigger risk and maybe even hamper the development of the seaweed sector, 30% answered “yes,” of which six were from industry stakeholders and two were from non-industry stakeholders. As justification, one respondent specified inorganic arsenic, while another specified PCBs and other chemicals. Another respondent explained that all hazards could be of higher risk if these are not monitored properly and that large-scale production could decrease or increase monitoring protocols. Consequently, these results further motivate investigation into which monitoring and/or mitigation measures are currently implemented, with hazards such as arsenic, iodine, and heavy metals considered as a relevant starting point.

The survey provided insight into the current monitoring measures or mitigation strategies that are being used by respondents. The following protocols were mentioned:

- Guide by Korean National Fishery Products Quality Management Service
- Protocols of good aquaculture practices
- Protocols of good manufacturing practices
- Hazard Analysis Critical Control Point (HACCP)
- Contamination risk assessment of harvest site (identifying potential sources of pollution, mapping exclusion zones)
- UK Governmental Standards
- Sample protocol by Wageningen Food Safety Research and NSF (presumed as North Sea Farmers)
- Legislation (European legislation on seaweed for food supplements and seaweed for feed: EC No. 1881/2006 and EC No. 2002/32, French legislation) CEN/TR 17559:2021—Algae and algae products—Food and feed applications: General overview of limits, procedures, and analytical methods
- Food Safety System Certification 22000 (FSSC 22000)
- British Retail Consortium (BRC)

In addition, some respondents answered that there is no need for new protocols or that they would not know what these new protocols should entail. A couple of other respondents urged for protocols that are more specific to the seaweed product (also for the seaweed species). Another respondent would like to implement HACCP, a food handlers’ certification, and a blockchain protocol. Two other respondents urged the development of legislation (EU and national), for example, by setting maximum levels for contaminants in food. One respondent mentioned a plastic detector as a new safety protocol. Another respondent explained the concern about the water quality regarding pesticides and pharmaceutical compounds and that a protocol for the monitoring of these hazards would be necessary.

Overall, according to the survey, relevant food safety hazards for the seaweed sector include arsenic, iodine, and heavy metals such as cadmium, lead, and mercury. Also, allergens may be of further interest to monitor given limited data combined with the potential health risk to certain consumers. Although the need for new protocols was questioned by the respondents, they did express the various needs for advice specific to the seaweed species, maximum levels, or detection matrix (plastic, water). Consequently, these results further motivate investigation into which monitoring and/or mitigation measures are currently implemented, with hazards such as arsenic, iodine, and heavy metals considered as a relevant starting point.

#### 3.2.2. Environmental Safety

Thirty-two respondents completed the environmental section of the survey and scored the twenty-two hazards. As for food safety, respondents were categorized into two groups, those working directly in the seaweed industry (*n* = 16) and those not working directly in the industry (*n* = 16).

Both industry and non-industry stakeholders scored the same three hazards as the top three risks: the input or spread of non-indigenous species other than cultivated seaweed, the input of genetically modified species and translocation of native species, and the release of productive material from domesticated seaweed species. However, respondents scored the top two in different orders. The input of genetically modified species was scored highest by industry stakeholders and second-highest by non-industry stakeholders, while the input or spread of non-indigenous species was scored highest by non-industry stakeholders and second-highest by industry stakeholders. A difference can be seen in the intensity of a risk which the two groups assimilated with a hazard. In general, non-industry scored the hazards as riskier than the industry group (Table 2).

The scores of the hazards, from highest to lowest, are similar in both groups, but there are a few exceptions with large variations in the scoring. The largest difference in scores can be seen for the “input or spread of non-indigenous species, other than cultivated seaweed.” The non-industry stakeholder group scored this a 4.0, with 25% of those respondents even rating it as a very big risk (5.0). From the industry stakeholder group, only one respondent (6%) rated it a very big risk (5.0), resulting in an average score from the industry stakeholders of 3.1. It is still seen in the top two hazards (Table 2). On the other hand, some hazards had the same score but had a different priority overall. For instance, “the introduction of non-synthetic substances,” such as heavy metals, hydrocarbons, etc., was 2.8 for both industry and non-industry stakeholders but ranked fourth overall for the industry stakeholder group and ninth overall for the non-industry stakeholder group. Similarly, the hazard “noise disturbance” scored 2.1 for industry stakeholders and 2.2 for non-industry stakeholders, but overall ranked 14th and 20th, respectively. This hazard belongs to the three hazards evaluated as the least risky by the non-industry stakeholders and shows the different perspectives these two groups have in terms of the level of risk these hazards pose. Overall, industry stakeholders evaluated over half of the hazards (59%) at 2.2 or lower, starting with as low as 1.6, while non-industry stakeholders only evaluated three hazards (14%) at 2.2 or lower, starting with 2.1. Unlike the riskiest hazards, there is no clear consensus on the least risky hazards.

When asked if the seaweed industry was to further develop in the future and if the hazards would then become a bigger risk and maybe even hamper the development of the seaweed sector, 12 out of 32 respondents (38%) mentioned there would be no further risk. Twenty respondents (63%) said that there are risks involved with a growing industry. They emphasized hazards such as “input of genetically modified species,” “shading,” “nutrient depletion,” “sedimentation,” “siltation,” and “change in the water flow” to have a growing impact on growing farm size. Others pointed out socio-economic issues such as “competing uses for waterways” or “objections from local populations.”

Respondents were also asked to name the standards or certification schemes that they mentioned to deal with hazards and reduce environmental impact. Those that responded indicated:

- Aquaculture Stewardship Council (ASC)
- B Corp and Tenure/Operation approval from Canadian government bodies
- Life Cycle Assessment (LCA)
- Organic certification
- Soil Association organic (for wild seaweed)
- Projects, or for example, the government and NatureScot assessing and monitoring environmental impacts

Another respondent stated that by only harvesting a third of the wild seaweed, sustainability and regrowth are assured.

In another question, the respondents were asked to name hazards that should be taken into consideration by the existing or new standards for environmental safety. The hazards, having been rated as a higher risk, were mentioned here again. “Non-native species introduction” and “Genetically modified organisms (GMOs)” were mentioned a few times, as well as littering, shading, and generally altering the ecosystem too much. The “introduction of any sort of synthetic or non-synthetic compounds” was also asked to be integrated into standards. Few respondents emphasized that all hazards should be subject to screening, explaining further that regulations should be focused on scale and proportionality rather than the blunt use of the precautionary principle. The concern of overregulating was also raised, saying that it could hamper the growth of the industry.

### 3.3. Interviews

Twelve seaweed companies were interviewed. Most interviews were with those persons directly involved with farming and/or harvesting seaweed, but a few companies were between the farming and processing parts of the value chain. The interviewed companies have locations in Europe (United Kingdom, France, Germany, the Netherlands, Faroe Islands, Spain, Norway, Ireland), as well as China, the United States, India, and/or Morocco. Usually, the founder, chief executive officer (CEO), or research scientist was interviewed. For the interviewed companies, the amount of seaweed farmed ranged from 0.15 tonnes of algae to 300,000 tonnes of fresh weight of seaweed, while the size of the companies ranged from 3 to around 3600 employees. The markets targeted included fresh, dried (including powders), and frozen seaweed for the food industry, among other markets like that for cosmetics and nutraceuticals. Most companies were looking to expand their yield and grow further in the food market, among other markets. A few emphasized the need to work with other research institutes or with other actors in the value chain to support their goals for growth. For each interview, the relevant hazards, as well as the monitoring and/or mitigation measures implemented, were identified.

#### 3.3.1. Food Safety

An overview of the food safety hazards and monitoring or mitigation indicated by the interviewees are shown per company (Table 3). The hazards identified were, among others, (pathogenic) bacteria such as *E. coli* and *Salmonella*, marine toxins, heavy metals (especially arsenic), minerals (especially iodine and zinc), and biofouling, allergens, and foreign body contaminants, such as that from ropes, plastics, or crustaceans, among others. Safety measures are currently taken by many of the interviewed participants. Mitigating strategies specified included harvesting according to good hygienic practices and from “clean” waters, testing, and using handbooks, working procedures, or protocols related to food safety. Blanching was noted to reduce iodine levels. For production environments, general good manufacturing processes, such as monitoring the temperature in the facility and where the product is transported or stored, were mentioned. Additionally, monitoring the microbial quality was indicated as a possible mitigation strategy.

Overall, according to the interviews, relevant food safety hazards for the seaweed sector include arsenic, iodine, and heavy metals such as cadmium, lead, and mercury. Also, pathogenic (bacteria) were mentioned by several interviewees. Mitigation strategies that follow good hygienic or manufacturing practices or other procedures or protocols were mentioned while monitoring microbial quality, product temperature, and blanching are currently practiced.

#### 3.3.2. Environmental Safety

Ten interviews addressed different environmental hazards, including both impacts from the farm on the environment (Table 4) and the environment on the farm (Table 5). Of the twelve companies interviewed, not all had answered each question, resulting in some companies not being shown in the tables (e.g., Table 4 shows seven companies and Table 5 shows ten companies).

Regarding the potential hazards where the environment has an impact on the farm, three companies mentioned their concerns regarding climate change, specifically the (increasing occurrence of) unpredictable and extreme weather. Other hazards that were identified concern contamination in the water, e.g., from ships, marine toxins, heavy metals, dead animals, and oil spills. Company four also identified biological hazards (bacteria, fungi, and viruses) as potential hazards. One company mentioned the occurrence of high temperatures, high humidity, noise, and dust in the production workplace.

Regarding the potential hazards caused by the farm that influence the environment, five companies mentioned ropes, nets, and other materials as possible sources of hazards. Such materials can shed microplastics into the ocean, get detached and pollute the ocean, or get detached and stuck in the propellers of ships. Furthermore, five companies identified the disturbance of aquatic life and marine habitats as a potential hazard. This was attributed to either noise pollution from boats or the (infrastructure of) cultivation sites themselves. Companies two and eight stated that the influence on aquatic life seemed to be positive so far, and Company four stated that more research needed to be done to determine whether seaweed farming has a negative effect on aquatic life. Several companies mentioned the potential of overharvesting or exhausting biomass in the environment. Freshwater usage and the energy-costly drying process were mentioned once by Company three. An adequate farm design was noted as a mitigation strategy against stones entering the vessel.

Overall, the interviewees mentioned marine toxins and climate change as the biggest risks for their farms, and as for the potential impact they might have on the environment, overharvesting and small microplastics from the ropes were their biggest concerns.

Preventive measures such as sustainable harvesting were mentioned by several companies as mitigation strategies to avoid harm to the environment through the farm. For instance, avoiding overharvest by harvesting in rotation or not cutting the root of the seaweed so that they can regrow. Furthermore, a couple of companies mentioned how harvesting with care avoids unnecessary harm, for instance, doing everything by hand. To avoid microplastics being shed from the ropes into the water or on the seaweed, farmers are waiting for biodegradable ropes, which are not yet on the market. To not disturb the natural habitat, farmers choose the sites carefully or avoid using substances such as antibiotics, pesticides, or other chemicals during cultivation and collect all visible little from the vessel.

## 4. Discussion

Our literature review identified more than 55 food safety hazards. Since it was impractical to have presented all possible chemical, physical, or microbiological hazards in the survey, the number of hazards shown in the closed section was reduced to 14. Although that may be seen as a limitation of the study, this was a conscious choice to prevent respondents from being overwhelmed or stopping the survey early. In turn, this helped focus the survey since the 14 hazards shown were selected on a more generic level by the hazard group. Moreover, open questions were included in the survey so that respondents could provide additional hazards or elaborate on their choices, and the interviews also allowed opportunities to further elaborate on key hazards. The seaweed industry is still in its infancy and has, compared to other aquacultures, a comparably low number of stakeholders involved. Nevertheless, the number of respondents to the survey (*n* = 36) is still too small to have a representative sample number to be able to draw general conclusions for the whole sector (according to the sample size determination table) [32]. Our survey also focused mainly on English-speaking respondents from Europe and North America. This may be another limitation of the study given a potential underrepresentation of, e.g., the Asian seaweed sector (*n* = 3). Nonetheless, this survey provides an indication and a comparison of the industry perspective on the safety of seaweed compared to other actors involved with seaweed.

### 4.1. Relevant Hazards

Our results have shown that there may be several types of food safety hazards associated with seaweed, with chemical hazards such as (inorganic) arsenic and iodine often reported. This result is unsurprising given the wealth of knowledge reported in the scientific literature and databases on these hazards in foods such as seaweed [10]. Inorganic arsenic is known to be the most toxic arsenic compound, and therefore, from a food safety perspective, it is important to monitor inorganic arsenic concentrations in seaweed intended for human consumption [33]. Inorganic arsenic is highly concentrated in some seaweeds, such as *S. fusiforme* (hijiki), in a way that one portion of 25 g would contain the same amount compared to the amount that a consumer would normally ingest via food in 1 to 2 months based on UK diets [34]. In general, brown seaweed species contain high concentrations of inorganic arsenic, followed by red seaweeds, while green seaweeds typically contain much lower concentrations [35]. Iodine is abundantly present in high concentrations in seaweed since iodine from seawater can be accumulated by seaweeds [36]. An explanation for the variation in iodine ranking between the industry stakeholder and non-industry stakeholder groups may be linked to differences in dietary guidelines and population exposure globally to iodine. Although it is an essential nutrient needed for biochemical processes in the human body, the amounts of iodine in seaweed may lead to an exceedance of the upper level of iodine for humans. Upper levels are established for nutrients, which the body requires for normal functioning, but can also exert adverse effects when the body is exposed to a high amount of the nutrient. This may lead to a risk of iodine surplus [19]. One gram of dried seaweed can contain more iodine than other food products made from terrestrial plants and can contribute between 20 and 500% to the recommended daily allowance of iodine for humans. Thereby, humans may be easily overexposed to iodine by the consumption of seaweed [37]. Children, pregnant women, and persons with thyroid dysfunctions are identified as specific subgroups that are at high risk for an iodine surplus [19]. Thus, inorganic arsenic and iodine are relevant food safety hazards across the seaweed value chain.

Besides these, other hazards such as cadmium, lead, and mercury (heavy metals), as well as pathogenic bacteria and allergens, have been noted [10,38]. This is reaffirmed by our literature review with its 55 food safety hazards.

When looking at hazards from an environmental perspective in the survey, “the introduction of non-synthetic substances”, such as heavy metals, hydrocarbons, etc., was also given importance by the respondents. The interview respondents also shared their concerns about contaminated water, e.g., from ships, marine toxins, heavy metals, dead animals, and oil spills. However, of those hazards having an impact on the farm, their biggest concern is the weather and rising temperatures due to climate change, which is also described by Chung et al. (2017) [39] as environmental challenges for seaweed aquaculture. Compared to the survey responses, in interviews, the focus was on publicly known issues such as microplastic shedding from the ropes, directly visible effects such as overharvesting in an area, disturbing animals or other industries by detached material such as ropes. In the survey, the highest-ranked hazards by the industry stakeholder group as well as the non-industry stakeholder group were microbial hazards such as the spread of non-indigenous or genetically modified species. This may be because the survey had a list of hazards to be ranked versus in the interview; there, it was posed as an open question or different aspects influencing if a hazard becomes a risk, which could not particularly be addressed in this study. In the reviewed literature, different aspects were considered and thus also led to various outcomes, although these align with the survey results as well as with the interviewees’ concerns. For example, according to Campbell et al. (2019) [22], the impact of the risk changes according to the size of the farm and should be assessed on a case-by-case basis. In this study, the release of reproductive material and the facilitation of disease, parasites, and non-native species, were ranked as a high risk, in line with the survey results. Yet, the addition of cultivation systems and the resulting potential pollution were evaluated as very low. In a different study on developing a framework and toolbox for measuring and evaluating ecosystem interactions of seaweed aquaculture (looking at the Dutch coastal zone and Delta waters), it was differentiated between instant or long-term high impact risk, low impact risk becoming nevertheless of relative importance when considering ecosystem components separately and even considering the impact on either lower trophic species or higher trophic species [14]. Here, the results are more in line with the interviewees’ concerns, ranking the potential impact of pressures such as the input of litter and introduction of non-synthetic substances and compounds with a high risk for higher trophic ecosystem components (i.e., fish, birds, and marine mammals). The introduction of non-indigenous species was ranked as a high risk for low trophic ecosystem components, but the input of microbial pathogens and parasites, or the input of genetically modified species, was given a low risk yet with importance due to the impact per ecosystem component. These different aspects could not be covered in our data collection. Furthermore, there are still big data gaps on the potential risks and their impacts, which makes definitive conclusions difficult [22,26,27,29,30,40,41]. Overharvesting is not mentioned as a hazard by the reviewed literature, as it refers rather to a harvesting practice.

### 4.2. Monitoring and Mitigation Strategies

Results showed that mitigations strategies aimed at preventing food safety hazards through good hygienic or manufacturing practices, food safety procedures or protocols, or pre-site farm selection while monitoring microbial quality, product temperature, and blanching are currently practiced. Many food safety-based protocols or guidelines are available or set up by food processors, although more frequently, these are on general food safety practices or are brought over from other food chains, such as aquaculture or fresh produce, as a starting point. The status of food safety public and private standards, including access to protocols, guidelines, or recommendations related to seaweed, have been previously discussed by Banach et al. (2020) [10]. The listed standards and certification schemes that help the farmers we reached in our study deal with environmental hazards or their farm’s impact on the environment from our results are reflected in the literature. However, our results also showed that there is still a need for further standards for environmental safety. Research on biosecurity aiming at controlling the spread and introduction of diseases, pests, and crops, as well as developing biosecurity policies and legislation managing the risks is required [12]. From our results, it can be seen that some stakeholders understanding the need to prevent hazards (e.g., with a pre-farm site check), while monitoring and reporting on (the change in) hazards such as arsenic, iodine, and heavy metals can be important to understand the potential risk. A review from Cavallo et al. (2021) [11] indicates a need to cultivate seaweed in a controlled environment with periodic checks on the finished product to control food safety hazards such as heavy metals. The need for controlled cultivation is a challenging prerequisite for non-land-based seaweed farming, where fluctuations in, e.g., the weather during the growing season may be difficult to predict or control. For example, Chung et al. (2017) [39] described the environmental challenges in the changing cultivation environment, e.g., through climate changes on a global level, when it comes to seaweed aquaculture. Yet, such effects are likely to be species-dependent, and much research is being invested in temperature-resistant species [42]. Our results reflected this lack of a solution for climate change-adapted species; cultivators mentioned not having any strategy for climate change effects, suggesting instead to cultivate on land and to use real-time monitoring for the local temperature rises [14,42]. Measuring the finished food product is not necessarily the most effective way to control food safety, although it can be a valid check for process and product verification [43]. Preventative approaches and food safety management systems may be a more effective approach for the seaweed sector to consider when dealing with microbiological food safety [43].

Besides relying on official regulations and certification schemes, environmental consciousness and a wish not to intervene in the environment were observed in our study. For example, using biodegradable material for ropes, avoiding the use or spilling of pesticides or chemicals, and only harvesting what can easily recover were at the forefront of their mitigation strategies. Literature supports this, naming preventative measures to avoid littering [14,22]. Furthermore, our study showed that the choice of the site for the farm can play a big role in the prevention of certain hazards, such as shading or nutrient depletion, which is in line with recommendations by literature [22,26,27].

Besides preventive measures, our results also showed that understanding that environmental hazards can become potential risks is part of a mitigation strategy. Biosecurity measures, together with combined monitoring and research to inform management strategies, the development of diagnostic techniques to rapidly detect diseases would be options to mitigate, for instance, the input on non-indigenous species or microbial pathogens [22].

### 4.3. Where Are the Gaps, and What Further Actions Are Needed?

New protocols and advice specific to the seaweed species, maximum levels, or matrix would be desirable to be able to optimize preventative strategies earlier on in the value chain. Protocols that consider a changing food system may help stakeholders understand the dynamics and future needs of the sector. Since monitoring measures and mitigation strategies specific to seaweed are less readily addressed in the scientific literature, a recommendation is to provide harmonized advice on monitoring and analyzing hazards in seaweed, such as arsenic or iodine, along with environmental hazards such as littering or the introduction of non-indigenous species, as that can help improve the risk assessment process.

In general, there is scarce data on the ecological effects directly linked to seaweed production, leading to deriving information from mussel cultivation to describe ecosystem interaction [22,44]. For the impact of the release of reproductive material, there is, therefore, no real data linked to seaweed farming. For better monitoring strategies, research to fill crucial knowledge gaps were already mentioned above; the reviewed literature further states that it is urgently required to assess the genetic resources of seaweed and develop adequate genetic conservation policies to be equipped to tackle the risk of introducing genetically modified species [29]. A strategic assessment of baseline conditions and breeding practices is also needed [14]. Avoiding the spread of non-indigenous species requires monitoring of good biosecurity practices by cultivators (e.g., biosecurity planning); more clarity regarding which target cultivation species are permitted is needed [22] as well as research to support adequate conservation policies [29]. There is no data to understand the effect fibers and microplastics could have on different ecosystem components [14].

The survey respondents had mentioned several certifications schemes and standards that help to keep the impact at a minimum; however, not enough data are available to adequately represent all hazards in such needed standards and protocols. Few respondents emphasized that all hazards should be subject to screening, explaining further that regulations should be focused on scale and proportionality rather than the blunt use of the precautionary principle. The concern of overregulating was also raised, saying that it could hamper the growth of the industry. Some respondents specifically mentioned development should only happen once these hazards are adequately understood and mitigated, and that impact depends on the regulations and adherence to them. Outreach and dissemination of information on seaweed, the benefit of seaweed intake, and the role of seaweed in regulating climate and water quality are needed to improve understanding in the sector.

## 5. Conclusions

This study showed that numerous hazards could occur in the seaweed value chain, from farm to fork, when considering its final use as a foodstuff. These were reported from food safety and environmental safety perspectives based on a scientific literature review, stakeholder interviews, and an expert survey. For food safety hazards, the findings were similar on the hazards to be considered as a concern, with relevant hazards including (inorganic) arsenic, iodine, and heavy metals, among others such as pathogenic bacteria. For environmental hazards, the findings were varied yet included environmental pathogens and parasites introduced into the ecosystem by domesticated seaweed, among others. These results may be due to extrinsic factors such as geographical differences at the cultivation site, but they may also be due to the scarce data on the intrinsic behavior of the hazards and their eventual impact on the product matrix or ecosystem. For instance, the impact and effects of the farm are expected and sometimes even visible, but very few studies show quantitative data on the consequences of these effects. Preventative measures can help avoid unknown consequences for the food safety of the seaweed as well as for the environmental safety of the ecosystem. Measures currently applied aim at preventing or mitigating hazards through good hygienic or manufacturing practices, food safety procedures or protocols, or pre-site farm selection, although the needs for the sector vary. General protocols to unify and standardize the information on monitoring and sampling, as well as best practice examples for mitigation, are needed for this growing sector. Altogether, developing protocols that align with the changing food system is recommended. This work could be complemented by applying this research to other sectors, not only the Chinese and European seaweed industries.

## Figures and Tables

**Table 1:** Survey scores regarding the risks for 14 food safety hazards in seaweed with 5: biggest risk and 1: smallest risk for hazards according to industry, non-industry, and all respondents.

Hazards	Industry Stakeholder (*n* = 16)	Non-Industry Stakeholder (*n* = 11)	Total (*n* = 27)
Chemical element—Arsenic	3.4	4.0	3.6
Chemical element—Iodine	2.6	3.4	2.9
Dioxins	2.7	2.4	2.6
Polychlorinated biphenyls (PCBs)	2.7	2.3	2.6
Physical—plastic	2.6	2.5	2.5
Pesticide residues	2.5	2.8	2.5
Bacteria—*Salmonella* spp.	2.4	2.3	2.4
Endocrine—disrupting compounds	2.4	2.0	2.3
Marine toxins (e.g., pinnatoxins, spirolides)	2.2	2.0	2.2
Pharmaceutical active compounds (e.g., antibiotics)	2.2	2.1	2.2
Bacteria—*Bacillus* spp.	2.3	2.0	2.2
Virus—Norovirus	2.4	1.7	2.2
Polycyclic aromatic hydrocarbons (PAHs)	2.2	1.5	2.0
Radionuclides	2.0	2.0	2.0
Average	2.5	2.4	2.4

**Table 2:** Survey scores regarding the risks for 22 environmental safety hazards in seaweed with 5: biggest risk and 1: smallest risk.

Hazards	Industry Stakeholder (*n* = 16)	Non-Industry Stakeholder (*n* = 16)	Total (*n* = 32)
The input of genetically modified species and translocation of native seaweed species.	3.5	3.9	3.7
The input or spread of non-indigenous species other than cultivated seaweed (if applicable).	3.1	4.0	3.5
The release of productive material from domesticated seaweed species.	2.9	3.4	3.1
The input of litter, for instance, by lost components.	2.7	3.2	2.9
The introduction of synthetic compounds, such as pesticides, antifoulants, and pharmaceuticals.	2.6	3.0	2.8
The introduction of non-synthetic substances, such as heavy metals, hydrocarbons, etc.	2.8	2.8	2.8
Shading; the absorption of light by the cultivated seaweed.	2.4	3.0	2.7
The attraction of species to the farm through the artificialization of habitat.	2.3	2.9	2.6
The input of microbial pathogens and parasites into the ecosystem by the domesticated seaweed.	2.4	2.6	2.5
Changes in siltation and sedimentation.	2.1	2.9	2.5
Entanglement of marine fauna in the cultivation structure.	2.1	2.6	2.4
Physical disturbance to the seabed.	2.1	2.6	2.4
The input of organic matter (DOM and POM *).	2.1	2.6	2.4
Extraction of or injury during harvest to wild non-seaweed species that are present around the seaweed farm.	2.1	2.4	2.3
The depletion of nutrients in the ecosystem by the domesticated seaweed.	2.0	2.5	2.2
Noise disturbance posed by anthropogenic sounds (for example, by boats and installation).	2.1	2.2	2.2
Collision of marine fauna with moving parts, such as a boat.	1.8	2.4	2.1
Visual disturbance of fauna posed by the farm structure.	1.9	2.4	2.1
Reductions in wave energy; caused by the absorption of kinetic energy by the farm structure.	1.8	2.4	2.1
Water flow changes; caused by the absorption of kinetic energy by the farm structure.	1.8	2.4	2.1
Extraction of a food resource by harvesting the seaweed.	1.9	2.1	2.0
The seaweed farm as posing a barrier to species movement.	1.6	2.2	1.9
Average	2.3	2.7	2.5

* DOM = Dissolved Organic Matter. POM = Particulate Organic Matter.

**Table 3:** Food safety hazards, the monitoring thereof, and measures to mitigate, as indicated by interviewees.

Company	Quality Monitoring and Tests	Main Hazards	Mitigation
1	End product, yearly, standard microbiological activity testing	Arsenic levels in Laminaria	Harvest from very clean water, which has been certified as grade A quality
		Poor water quality	-
2	The end product is monitored via microbiological tests (maybe 2–3 times a year) and heavy metal tests (once a year).	-	Following sanitary regulations, washing leaves by hand.
	The regional government monitors the water in which the seaweed grows, e.g., on toxins (multiple times per year, maybe even every week).	-	-
3	Samples are taken daily when it comes in and are tested on heavy metals, bacteria (*Escherichia coli*, *Salmonella*), and marine toxins.	Heavy metals, bacteria (*E. coli*, *Salmonella*), and marine toxins.	-
	After the processing step of washing, the product is tested on chlorates. Semi-finished products and final products are also tested.	-	-
4	Each batch of fresh produce and right after the last processing step on heavy metals, minerals (e.g., iodine, zinc), and pathogens.	Heavy metals, minerals (e.g., iodine, zinc), pathogens, fouling.	-
5	Water samples are taken to check on *E.* *coli*.	Biofouling, snails	Washing with fresh water
	Every batch (end product) is tested for seven parameters (*E. coli*, *Listeria*, etc.) internally and once a month externally.	*E. coli*, *Listeria,* and other bacteria	Testing, food safety handbook
		Arsenic	-
6	Seaweed is tested when it comes into the processing facility.	Heavy metals (e.g., cobalt) and iodine.	-
7	Biomass in the sea during the growing phase every 2–4 weeks (biofouling).	Biofouling, animals attached to the seaweed, iodine, degradation of the fresh seaweed quality.	Test a lot (see the column for quality monitoring), blanching to reduce iodine levels.
	Microbial composition (one sample of each production day, pooled and tested).		
	Chemical composition for the end product (protein, heavy metals, minerals, other basics).	-	-
	Quality control of the process once per season.	-	-
8	The macroalgae are monitored by the client before they make their products; therefore, some data is available on iodine content and heavy metals. The farm does not monitor.	High content of iodine and arsenic in the sugar kelp.	Respondent did not mention a specific mitigation strategy; algae are currently not used as food; quantities are too low
	The water is not monitored specifically for the macroalgae. The mussels in the water are monitored weekly during the harvest period. Since they absorb the water, this also tells something about the safety of the water.	-	-
9	For culturing environments, nutrient concentrations (nitrogen, phosphate) are monitored monthly; temperature and salinity are monitored daily.	With monitoring closely, there is no major concern for safety or quality. Storage for overstocking is a potential challenge under hot weather, but the current status is in a demand-and-supply balanced or demand-exceeds-supply status.	Equipped with cold storage with a capacity of 100,000 tons.
	For the production environment (workplace), temperature and humidity are monitored in real-time.		
	For end products, parameters listed in the national and industrial standards are tested based on daily batch sampling, including heavy metal concentrations, water and salt content, microorganisms, appearance, etc.	-	-
10	For the production environment (workplace), temperature, humidity, noise, and dust are monitored in real time.	Spoilage in high temperatures or on rainy days, particularly when humidity >60% or temperatures reach 28 °C.	Equipped with cold air system, ventilator, and air conditioning system.
	For products, parameters listed in the national and industrial standards are tested based on daily batch sampling, including heavy metal, purity, bacteria, water, salt content, etc.	-	-
11	Products for the domestic market follow national and industrial standards of food safety and seaweed products, including heavy metals, microorganisms, etc. Exports follow the local standards of importing countries.	Spoilage may happen when humidity is high.	No truck loading on humid days.
12	Monitor microbiology (safety and spoilage), heavy metals, iodine, and some screening on polycyclic aromatic hydrocarbons (not 100% testing).	Iodine, foreign body contamination, allergens.	Process (heat) to eliminate or reduce metals. Monitor microbiological quality. Developed own working procedures/protocols for food safety and comes with a buyer specification sheet (for farmers).

A dash (-) means no information was provided.

**Table 4:** Environmental hazards that have an impact on the farm.

Company *	Hazards	Mitigation
1	Climate change: unpredictable weather with high waves and storms; rising the sea level	Grow seaweed on land (future); the design of the farm should not be so dependent on nature
	Contamination risk (dead animals, oil spills, farm runoff) is a potential hazard but not a worry	
4	Ships can introduce contaminants into the farm.	n/d
	Ships can (mechanically) damage the far	n/d
	Marine toxins	n/d
	Biological hazards (bacteria, fungi, viruses)	n/d
7	Stones	Farm design
	Heavy metals from runoff from the agricultural industry	
8	Contamination in the water: limited because the farm is located next to the biggest harbor police station/flow of the water. Shipping routes are close by, but ships never enter the area of the farm due to the presence of the military in the past.	
	Escape of fish from other farms	No mitigation strategy (“waiting on what happens”; vulnerable in such scenarios)
9	Climate change: extreme weather with strong winds and high waves	No particular mitigation strategy.
10	High temperature (28 °C), high humidity (>60%), noise, and dust in the production workplace	Real-time monitoring
11	Climate change: extreme weather with strong winds and high waves	

* Only the companies who responded to the question of possible hazards and their mitigation strategies. n/d.: no data.

**Table 5:** Environmental hazards where the farm has an impact on the environment.

Company *	Hazards	Mitigation
1	Ropes shed microplastics	Everything is done by hand to have a low impact.
	Noise pollution from the boat	For the ecological certificate in Norway, they have to recycle the ropes because they cannot re-use them.
	Potential effect: overharvesting	Avoid overharvesting: harvest in rotation, only cut part of the plant
2	Potential effect: Disturbance of aquatic life due to concrete blocks on the bottom of the sea. However, currently, the blocks seem to have a positive effect since the cultivation area attracts many fish.	Blocks were placed on the sand
	Nets and other materials can pollute the sea.	Being careful with materials, divers usually are.
	Potential effect: overharvesting	There are regulations in place that specify how to harvest seaweed, e.g., seaweeds are cut so that the root can stay, and seaweeds are not harvested year-round. A part of the seaweed population is NOT harvested, except for seaweed species that are not natural to the area (the government wants to get rid of these species). The company buys from fishermen who are under [environmental] control of the government.
3	Exhaustion of biomass in the environment, arsenic in the environment, freshwater usage, energy-costly drying processes.	Sustainability planner, right harvest tools, purification of processing water, re-use of freshwater, use of waste streams to generate energy.
4	Possibly breach of marine biodiversity	More research is needed if this is a real hazard.
5	Very small risk of spillover from oil products from the boat	
	Equipment (ropes) is not fit for the weather conditions, and getting detached or breaking off represents a high risk for other industries on the water.	Precaution that all equipment withstands the extreme weather conditions.
	Plastic	Nylon is used
6	Disturbance of natural marine habitat.	Cultivation sites are carefully selected.
7	Nano and microplastics from ropes	
	The fishing industry is worried that they will be negatively affected by the farm	
8	(Micro)plastics that accumulate in the water because of the ropes used during farming.	
	Detachment of ropes/lines could get stuck in the propeller of ships	Use of strong ropes
	Potential effect: Disturbance of aquatic life due to concrete blocks on the bottom of the sea. However, currently, the blocks seem to have a positive effect since fishermen can’t come near the blocks (‘retreat for fish’)	n/a
	Sedimentation levels and oxygenation levels are higher underneath the farm because of the permanent rain of nutrients going down from the mussels.	n/a
9	They indicate no impact on the environment	No antibiotics, pesticides, or other chemicals are used during cultivation; all trash and facilities are collected and taken back to land after use in the field.
10	They indicate no impact on the environment	
11	They indicate no impact on the environment	n/a

* Only the companies who responded to the question of possible hazards and their mitigation strategies. n/a: not applicable. n/d.: no data.

## Data Availability

The data presented in this study are available on request from the corresponding authors. The data are not publicly available due to confidential data in the interviews and survey responses.

## Supplementary Materials

The following supporting information can be downloaded at: https://www.mdpi.com/article/10.3390/foods11101514/s1, Supplementary Materials—Interview Guide.
