# Supplementary material for: Seaweed Value Chain Stakeholder Perspectives for Food and Environmental Safety Hazards
Source: Foods. 2022 May 23;11(10):1514. doi: 10.3390/foods11101514 (PMC9141909; doi:10.3390/foods11101514)
Supplement: Supplementary file 1 [file foods-11-01514-s001.zip › foods-1674498-supplementary.pdf]

Research paper: Supplementary Materials

Seaweed value chain stakeholder perspectives for food and environmental safety hazards

J.L. Banach [1a*], S. Koch[2a], Y. Hoffmans [1], S.W.K. van den Burg [2]

[1] Wageningen Food Safety Research (WFSR), Wageningen University & Research, 6700 AE Wageningen, the Netherlands; jen.banach@wur.nl; yvette.hoffmans@wur.nl
[2] Wageningen Economic Research, Wageningen University & Research, Droevendaalsesteeg 4, 6708 PB, Wageningen, the Netherlands; sophie.koch@wur.nl; sander.vandenburg@wur.nl

* Correspondence: jen.banach@wur.nl (J.L. Banach); Tel.: +31 614 323 125
[a] These authors contributed equally to this work.

Supplementary Materials

Interview guide

Part A. General questions

1. Can you briefly introduce your seaweed company to us? (What is your role?)
2. How do you view your future business? How are food safety, occupational health & safety, and environmental safety included?
3. What are your production volumes?
4. What are your final products? What form are these? (wet or dry)
5. For which market(s) do you produce seaweed for food?

Part B. Specific questions on hazards (food safety and environmental)

Food safety and perception of hazards

1. How would you describe the quality of your production over the season(s)?
2. How do you monitor the quality of where your product is produced?
    a. On what do you monitor/What is tested?
    b. What is the frequency of monitoring?
3. How do you monitor the quality of your product?
    a. On what do you monitor/What is tested?
    b. What is the frequency of monitoring?
    c. In which phases of your product cycle/life is it monitored? (Think of unharvested, right after harvest, after first processing step, finished product.)
4. Can you name any problems/concerns that you have, e.g., possible contamination or rapid spoilage of your product?
5. Under which specifications are your product produced/marketed?
6. What private (food) standards do you currently follow or seek to follow in the coming 1-2 years?
7. How is your product packed (and in what material), and how is it transported?

Environmental safety

1. Can you tell us more about any environmental hazards that are relevant in seaweed farming? (both current hazards and potential hazards/ask more about causes/per hazard also ask about the impact on value-chain either (i) a hazard where a seaweed farm has an impact on the surrounding environment or (ii) a hazard from the environment that has an impact on the farm)
2. You did not mention the following factors ______ Can you elaborate a bit on their influence?

   Hazards regarding (i)

   a) Mega)fauna mortality, b) (Mega)fauna disturbance, c) Excessive nutrient absorption and removal, d) Pollutions, e) Changes in hydrodynamics, f) Prevalence of diseases, g) Genetic depressions of natural algal population, h) absorption of light, i) artificial habitat creation, j) Release of particulate and dissolved organic matter

   Hazards regarding (ii)

   k) contamination from close by wastewater, l) marine biotoxins, antibiotics, m) possible oil spills, n) dead animals (possible increase with windmills around) that contaminate the seaweed due to bones, bacteria, fungi, and viruses o) The release of materials from substrates and equipment p) microplastics
3. Which environmental safety measures do you know of that can be taken to minimize the effects of [hazard]? [In case of producer] Which safety measures do you currently take to minimize the effects of [hazard]?

<u>Part C. Questions on what the company needs and/or likes to have</u>

1. Do you have any thoughts on improvements/innovations that you need?
2. Can you name any tools/solutions/improvements/innovations that you (desperately) need to improve your product's quantity and/or quality?

3. Do you see possibilities and/or hurdles for inserting an additional processing step in your production process?

4. How could, in your daily practice, any possible safety hazards be monitored feasibly, according to you?

5. What do you expect to achieve in the coming 5 years in your company in terms of turnover, innovations, developments for production, or seaweed products?
